# Approaches for Studying Autophagy in *Caenorhabditis elegans*

**DOI:** 10.3390/cells6030027

**Published:** 2017-08-30

**Authors:** Yanfang Chen, Vincent Scarcelli, Renaud Legouis

**Affiliations:** Institute for Integrative Biology of the Cell (I2BC), CEA, CNRS, Univ. Paris-Sud, Université Paris-Saclay, 91198 Gif-sur-Yvette CEDEX, France; yanfang.chen@i2bc.paris-saclay.fr (Y.C.); vincent.scarcelli@i2bc.paris-saclay.fr (V.S.)

**Keywords:** *C. elegans*, genetics, in vivo imaging, electron microscopy, mitophagy, aggrephagy, LGG-1, LGG-2

## Abstract

Macroautophagy (hereafter referred to as autophagy) is an intracellular degradative process, well conserved among eukaryotes. By engulfing cytoplasmic constituents into the autophagosome for degradation, this process is involved in the maintenance of cellular homeostasis. Autophagy induction triggers the formation of a cup-shaped double membrane structure, the phagophore, which progressively elongates and encloses materials to be removed. This double membrane vesicle, which is called an autophagosome, fuses with lysosome and forms the autolysosome. The inner membrane of the autophagosome, along with engulfed compounds, are degraded by lysosomal enzymes, which enables the recycling of carbohydrates, amino acids, nucleotides, and lipids. In response to various factors, autophagy can be induced for non-selective degradation of bulk cytoplasm. Autophagy is also able to selectively target cargoes and organelles such as mitochondria or peroxisome, functioning as a quality control system. The modification of autophagy flux is involved in developmental processes such as resistance to stress conditions, aging, cell death, and multiple pathologies. So, the use of animal models is essential for understanding these processes in the context of different cell types throughout the entire lifespan. For almost 15 years, the nematode *Caenorhabditis elegans* has emerged as a powerful model to analyze autophagy in physiological or pathological contexts. This review presents a rapid overview of physiological processes involving autophagy in *Caenorhabditis elegans*, the different assays used to monitor autophagy, their drawbacks, and specific tools for the analyses of selective autophagy.

## 1. *C. elegans*, an Animal Model to Study Autophagy

### 1.1. General Experimental Advantages

A *Caenorhabditis elegans* adult is an approximately 1 mm, transparent nematode, predominantly found as a self-fertilizing hermaphrodite. *C. elegans* males emerge spontaneously at low frequency and are able to fertilize hermaphrodites, which allows for all genetic approaches. The life cycle of *C. elegans* includes an embryonic phase, four consecutive larval stages (L1–L4) separated by cuticle sloughing, and the adult stage, during which the worm is reproductively mature. In favorable conditions, the lifespan of wild-type *C. elegans* is approximately two to three weeks. In response to harsh environments, early larvae can enter an alternative larval stage called dauer, which enables them to survive for several months. *C. elegans* has a rapid development, three days, and is highly fertile, with a progeny of a few hundred for a single hermaphrodite. Its cell lineage, 959 somatic nuclei in the adult hermaphrodite, is invariant, and its stereotypical development leads to the differentiation of various cell types and tissues (epidermis, intestine, muscle, neurons…). Its small size and transparency are convenient for diverse microscopy approaches, and populations can be synchronized and cultured in liquid medium to obtain a large quantity of biological material. Classical genetic tools are available in *C. elegans,* and transgenesis or RNA interference (RNAi) are commonly performed. Moreover, the genomic engineering by CRISPR-Cas9 has been recently adapted to *C. elegans* to efficiently create new transgenic or mutated strains [1]. All these experimental advantages established *C. elegans* as a good model, which has been key in the understanding of major biological processes such as the apoptotic cell death or the discovery of RNAi.

### 1.2. Physiological Processes Involving Autophagy in C. elegans

The *Atg* (autophagy related) genes, which are involved in various aspects of autophagy, were first identified in yeast, but are mostly conserved in other eukaryotes (Table 1). In mammals, numerous orthologs emerged from genetic amplifications, but in *C. elegans*, most *atg* genes have one single ortholog. However, two homologs exist for *Atg4*, *Atg8,* and *Atg16* (Table 1). Interestingly, each homolog of the ubiquitin-like protein ATG8, named LGG-1 and LGG-2, corresponds to a specific member of the GABARAP and LC3 families, respectively. Both LGG-1 and LGG-2 are conjugated to a phosphatidylethanolamine (PE) lipid present in the membrane of phagophore and autophagosome [2]. Although *lgg-1* is essential for development and fertility, *lgg-2* null mutants do not present a gross developmental or morphological phenotype. In regards to the limited genetic duplications and the simplicity of the animal, *C. elegans* has emerged as a powerful alternative model for exploring autophagy in the context of tissue-specificity and developmental processes [3,4,5].

During the last 15 years, numerous studies on *C. elegans* have shown that autophagy is involved in multiple processes through embryonic and larval development. Upon fertilization, the degradation of several paternal organelles is dependent of the formation of autophagosomes. This fast process, called allophagy (allogenic organelles autophagy), leads to the degradation of the sperm components by selective autophagy, and is linked to the polyubiquitination of these organelles [19,24]. In this particular process, it has been shown that LGG-1 acts upstream of LGG-2, with LGG-1 being involved in the early steps of autophagy, and LGG-2 in the maturation of autophagosomes [25]. Later during embryogenesis, a selective autophagy process called aggrephagy is involved in the removal of proteins that are prone to form aggregates (see below Section 3.1.1). During this process, different maternally inherited germline-specific components are selectively eliminated by aggrephagy in somatic cells, allowing their restriction to the germline precursor cells. The depletion of various autophagy proteins results in a late embryonic lethality demonstrating that autophagy also plays a role in differentiation and organogenesis [30]. During larval development, autophagy acts as a response to unfavorable environmental conditions. For instance, autophagy allows newly hatched L1 larvae to survive one to two weeks without nutrients [20]. Autophagy also has a critical role for the epidermal differentiation of the dauer larvae stage, which enables survival in harsh environments for several months [31]. 

Autophagy is also involved in the response to various kinds of stress such as osmotic stress, oxidative stress, starvation, and resistance to pathogens. The inhibition of autophagy in *C. elegans* leads to a reduced survival under particular stress conditions [20,21,32,33,34]. For instance, inactivation by RNAi of autophagy genes (*atg-7*, *bec-1*, *lgg-1*) decreases the survival rates in *S. thyphimurium*-infected animals and suppresses the resistance conferred by the insulin-like signaling pathway. Hormetic heat stress induces autophagy, and thus improves survival and proteostasis in the nematode [35].

Using genetic contexts where the lifespan of the worm is extended (*eat-2*, *daf-2*, *let-363*, among others), studies have shown that aging and longevity are dependent on autophagy [16]. Indeed, the long-lived phenotype can be suppressed when several autophagic genes are inactivated (*lgg-1*, *atg-18*, *bec-1*, *atg-7*, *atg-9*, *vps-34*) [36]. Several pieces of evidence link autophagy and longevity in *C. elegans* [16,23], notably through the insulin pathway, which is consistent with knowledge on other species.

Cell death is crucial for many aspects of normal development, and can occur by apoptosis, necrosis, or other processes. *C. elegans* is a well-established model to study cell death, and more than 10% of cells undergo programmed cell death during embryonic and larval development [37]. Autophagy genes are involved in apoptotic cell degradation during development, and in germline cells. Dying cells lead to the formation of cell corpses, and autophagy proteins (BEC-1, UNC-51, ATG-18) were shown to be involved in the removal of those corpses in germline cells. In addition, the inactivation of *bec-1*, the ortholog of human Beclin-1 that interacts with the apoptotic factor CED-9/BCL2, causes an augmentation of the number of apoptotic cell corpses in embryo [38]. Moreover, some classical autophagy proteins are implicated in the so-called LC3-associated phagocytosis (LAP), which is involved in apoptotic cell removal. LAP is distinct from autophagy because it does not require the whole autophagy machinery, and LC3 is recruited to a single membrane vesicle. BEC-1 is critical for the removal of cell corpses in germline cells by a LAP process. Recently, a LAP-dependent degradation, requiring BEC-1, LGG-1 and LGG-2, but not UNC-51 and EPG-8, has been demonstrated for mid-bodies in *C. elegans* embryo [39]. Using a model of necrosis for touch neurons, some studies have shown that autophagy is upregulated in early phases of necrotic cell death, and synergizes with the lysosomal pathway [18,40].

Until 2016, the relationship between autophagy and tumor cells has not been addressed using available models of *C. elegans*. However, a first report demonstrated that the upregulation of autophagy has a role in limiting the growth of heterogeneous tumors in the gonad [41].

## 2. Methods to Monitor Autophagy in *C. elegans*

### 2.1. Imaging Autophagy

One of the most common ways to monitor and study autophagy consists of imaging autophagic structures, receptors and cargoes. The transparency of *C. elegans* has facilitated the development of light microscopy approaches, either in vivo or on fixed material. Additionally, different protocols have been optimized for analyzing autophagy by electron microscopy in *C. elegans*.

#### 2.1.1. In Vivo Imaging

**Figure 1:**
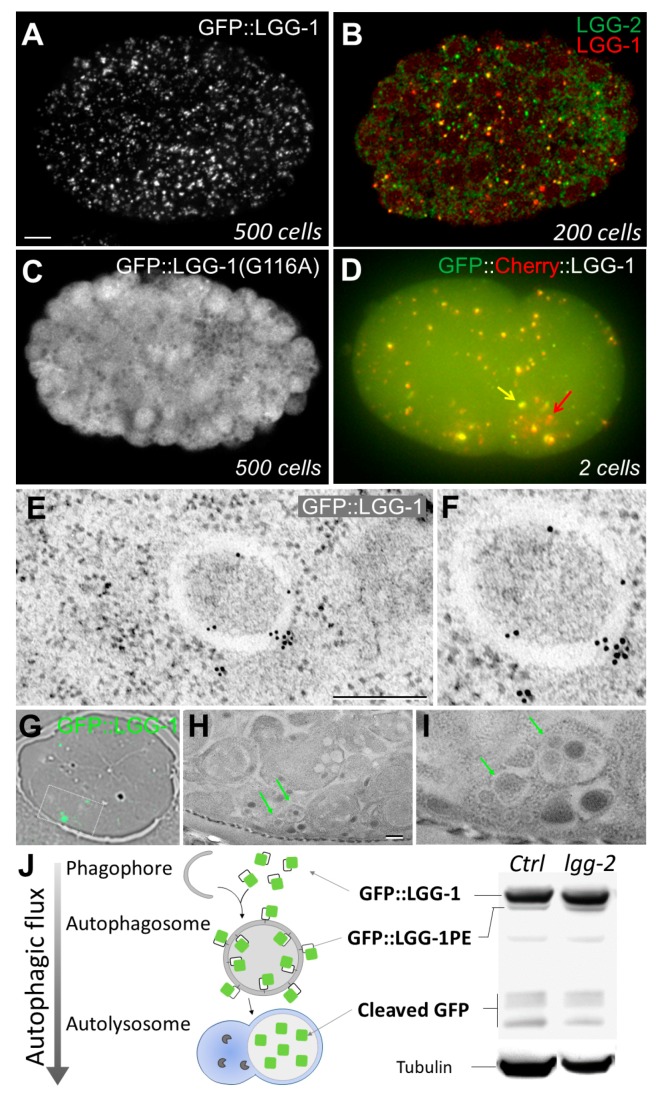
Use of LGG-1 and LGG-2 to monitor autophagy in *C. elegans*. (**A**) In vivo confocal picture of GFP::LGG-1 in a 500-cell embryo. GFP::LGG-1 puncta correspond to autophagosomes; (**B**) Merge confocal picture of a co-immunostaining of LGG-1 and LGG-2 in 200-cell embryo. Three types of puncta can be distinguished: LGG-1 positive (red), LGG-2 positive (green), and double positive (yellow); (**C**) In vivo confocal picture of GFP::LGG-1(G116A) form in a 500-cell embryo. This non-lipidated form presents a diffuse localization pattern with no puncta; (**D**) Epifluorescence merge picture of the tandem GFP::mCherry::LGG-1 in a two-cell embryo. The yellow arrow indicates an autophagosome (yellow resulting from GFP and mCherry fluorescences), whereas the red arrow shows an autolysosome (only mCherry fluorescence in acidic compartment); (**E**,**F**) Electron micrographs of GFP::LGG-1 embryos incubated with antibodies coupled to gold beads, revealing autophagosomal structures; (**G**–**I**) Correlative light and electron microscopy (CLEM) analysis of GFP::LGG-1; (**G**) Merge between bright field and fluorescence images of an ultrathin section of an embryo; (**H**,**I**) Electron micrographs of the boxed region in G. Green arrows indicate GFP::LGG-1 positive autophagosomes; (**J**) Schematic representation of autophagic flux and Western blot analysis of GFP::LGG-1 using GFP antibodies. The cleaved GFP correspond to a product of degradation in autolysosomes. The relative quantity of GFP::LGG-1 and the phosphatidylethanolamine (PE) conjugated form allows the measurement of the autophagic flux. Tubulin is used for normalization. Scale bar: 5 µm (**A**); 2 µm (**E**); 1 µm (**H**).

Similarly to Atg8/LC3, which are widely used to monitor autophagy [42], LGG-1 and LGG-2 are the most frequent markers to localize autophagosomes in *C. elegans*, because they are recruited to autophagosome through the lipidation of their C-terminus. Several strains expressing fluorescent fusion proteins with LGG-1 and LGG-2 are available (Table 2). In 2003, Melendez and colleagues generated the first strain expressing GFP::LGG-1, driven by its own promoter [31], which rapidly became the most common way to localize autophagosomes in *C. elegans* (strain DA2123 [20])*.* GFP::LGG-1 shows a dynamic pattern of dots during embryogenesis, which appears along with a diffuse signal in some cells that can be reduced by confocal imaging (Figure 1A). In adult and larva, in nutrient-rich conditions, GFP::LGG-1 is diffused in various tissues. Upon starvation, the number of GFP::LGG-1 dots increases mostly in hypodermis, seam cells, and intestinal cells, corresponding to an activation of autophagy [4]. The quantification of autophagy has been performed by counting GFP::LGG-1 dots at most stages of development and in most tissues. However, care should be taken, since some cells present a high cytosolic diffuse signal, and because GFP::LGG-1 is a multiple-copies transgene with an overexpression that might affect the number of autophagosomes, and possibly forms aggregates in particular conditions. Moreover, despite an expression under LGG-1’s own promoter, DA2123, GFP::LGG-1 is silenced in the germline and the very early embryo. The use of a stage-specific promoter, *Ppie-1*, has overcome this difficulty (strain RD204 [25]). DsRed::LGG-1 and mCherry::LGG-1 have been generated, although they have been less extensively used and are more prone to aggregation, especially in nutrient deprivation conditions [5,20,43]. Interestingly, a tandem fusion GFP::mCherry::LGG-1 has been created, which allows for the monitoring of the autophagic flux in vivo (Figure 1D) [25]. GFP and mCherry have different sensitivities to the acidic pH of the lysosome, which enables autolysosomes (mCherry-only positive dots) to be distinguished from autophagosomes (GFP and mCherry positive dots). However, due to the *pie-1* promoter-driven expression, this tool can only be used in the early embryo and germline cells [25].

LGG-2 has been less characterized than LGG-1, but GFP::LGG-2 can also be used as an alternative marker to monitor autophagy activity during development, because its localization at phagophores and autophagosomes has been validated by immunoelectron microscopy [25]. Of note, two particular constructs have been generated, GFP::LGG-1(G116A) and GFP::LGG-2(G130A), which inhibit their lipidation and can be used as controls to exclude aggregation artefacts (Figure 1C).

Another caution about the interpretation of LGG-1 and LGG-2 patterns arises from recent studies that show that in starved mammalian cells, the LC3 family is dispensable for autophagosome formation [44]. This finding supports the idea that an autophagosomal-like structure can be formed in the absence of the Atg conjugation system [45]. Thus, it is possible that LGG-1 and LGG-2 may not be involved in all autophagy processes. To overcome these potential limitations, alternative and reliable markers of autophagosomes should be investigated in *C. elegans*. One interesting possibility would be to use the syntaxin 17 (STX17), which labels closed autophagosomes in mammals [46]. Unfortunately, there is no obvious homolog of *stx17* in the *C. elegans* genome. Alternatively, the identification of other *C. elegans ATG* orthologous genes enabled the generation of several tools to visualize autophagy in vivo. Fluorescent reporters such as GFP::BEC-1, GFP::ATG-4.1, GFP::ATG-9, and GFP::ATG-18 have been generated, and their specific expression patterns have been described [2,6,38,47]. Those reporters have not been frequently used, but should emerge as powerful complementary tools to further visualize and analyze the autophagy process, especially for early steps of phagophore formation. Additionally, the analysis of aggrephagy by Zhang et al. led them to develop several interesting tools [4,7], which are not restricted to this particular autophagy process. For instance, several GFP-tagged proteins can be used to analyze the degradation or visualize specific cargoes, such as the SEPA family and the autophagy receptor SQST-1 (see Section 3.1).

#### 2.1.2. Immunostaining of Autophagy Proteins

Additionally to fluorescent reporters, antibodies against several autophagy proteins have been produced and used in *C. elegans* (Table 2). Immunostaining of the endogenous LGG-1 and LGG-2 proteins revealed dots that are similar to the GFP::LGG-1 dots in transgenic worms [6,24]. While GFP::LGG-1/LGG-2 reporters are useful for observations of live animals, immunostaining is essential to reflect the endogenous protein localization. Anti-LGG-1 and LGG-2 antibodies confirmed that the basal level of autophagy is not constant during embryonic development, with a maximum puncta between 100-cell and 200-cell stages [4]. In different autophagy mutant contexts, the pattern of LGG-1 is drastically modified. For instance, in *atg-3* and *atg-7* mutants, both required for LGG-1 conjugation to PE, no LGG-1 puncta are detected [9]. In *atg-2*, *atg-18* and *epg* mutants, the progression of the autophagy flux is impaired, resulting in the accumulation of LGG-1 puncta [6,9]. Co-localization approaches using anti-LGG-1 and anti-LGG-2 antibodies allowed researchers to highlight the existence of three distinct populations of autophagosomes: LGG-1 only, LGG-2 only, or double positive (Figure 1B) [25]. The co-localization approaches between LGG-1/LGG-2 and other proteins, for instance the autophagy substrate SEPA-1, are easy to perform by immunostaining in the embryo, but this approach is more complicated in larva or adult, especially for some tissues. Because anti-LGG-1 and anti-LGG-2 antibodies are limited resources and not commercially available, some studies used anti-GFP and anti-mCherry antibodies in reporter-expressing strains to perform co-localization analyses. 

#### 2.1.3. Electron Microscopy

At the end of 1950s, electron microscopy (EM) allowed researchers to observe double-membrane vesicles, which were first coined as “autophagosomes”, by C. de Duve in 1963 [49,50]. The three main steps of the autophagy flux were described first on a morphologic basis: phagophore, autophagosome, and autolysosome. EM is still considered to be one of the most reliable approaches in the study of autophagy, as it allows the anatomic observation of the different autophagic structures without labelling. It is also a very unique method for analyzing many detailed aspects, from the sub-cellular compartments to the tissue organization when autophagy is impaired. EM approaches have been applied and optimized for *C. elegans* [51]. When *C. elegans* is grown in starvation conditions, it has been observed through EM that the relative volume of autophagic structures increases 10-fold [34]. Autophagic structures have also been shown by EM analysis to accumulate under oxidative stress, and in mutants such as *glp-1* and *daf-2* [17,31]. In the *epg-3* or *epg-4* mutants, an abnormal accumulation of phagophore is observed, which supports the idea of a role for these proteins in the early steps of autophagosome formation [9]. Therefore, the anatomic analysis of autophagic structures by EM, combined with a quantitative analysis, allows for a good characterization of the autophagy flux. Moreover, combining EM with gold immunostaining can create a more specific analysis of various autophagy proteins or processes. Indeed, the presence of LGG-1 and LGG-2 at the membranes of phagophores and autophagosomes were confirmed with this technique (Figure 1E). As an alternative approach to immunogold labelling, correlative light and electron microscopy (CLEM) allows researchers to study the distribution of an autophagy protein when antibodies are not available. The principle is to combine EM with the observation of the fluorescence of a reporter by using fixation conditions and embedding resin that preserves the fluorescence. Such a technic has been efficiently used for GFP::LGG-1 and GFP::LGG-2, allowing researchers to correlate fluorescent puncta with vesicular structures (Figure 1G) [25].

EM-based approaches present the advantages of being anatomical, very resolutive, and independent of any markers, so they are often essential to further confirm light microscopy observations. Although EM approaches are very informative, they can only be performed on fixed tissue, which can generate artefacts; they are time and resources consuming; and they require a high level of expertise. Moreover, rare events could be very difficult to identify, and 3D reconstitutions are complicated to perform in routine. Therefore, EM and light microscopy analysis are very complementary approaches.

### 2.2. Molecular Approaches

Autophagy is a very dynamic process whose regulation relies on multiple parameters. Although imaging autophagy reveals insights about important features of the process, it is not sufficient to address all aspects. In order to monitor the level of autophagy and/or the completion of the different steps of the autophagic process, different molecular tools are available that enable the quantitative analysis of protein modifications.

#### 2.2.1. Monitoring Autophagic Flux by Western Blotting

LGG-1 is synthetized as a precursor protein and immediately cleaved at position 116 by the protease ATG-4.1 to generate LGG-1-I, a protein diffuse in the cytosol with a C-terminal exposed glycine residue. By a conjugation mechanism involving several ATG proteins, LGG-1-I is lipidated to PE, thereafter called LGG-1-II, and associated at the membrane of the phagophore and autophagosome [25]. Due to their differences in molecular weight and lipid modification status, LGG-1 forms can be separated on a SDS-page gel, with LGG-1-II migrating slightly faster than LGG-1-I, and revealed by Western blotting (WB). The relative quantity of the LGG-1-II form compared with LGG-1-I can generally be correlated with the number of autophagosomes. Measuring the ratio of LGG-1 conjugated to autophagosomes is a way to quantify autophagy activity in different contexts [4,52]. For example, in embryos, the LGG-1 precursor is not visible, and LGG-1-II is in small minority compared with LGG-1-I. When autophagy flux is blocked, either during autophagosome formation or maturation (*epg-3*, *epg-5*), both LGG-1-I and LGG-1-II levels increase. In contrast, when LGG-1 cleavage is impaired (*atg-4.1* mutant), a significant accumulation of LGG-1 precursor is observed, and LGG-1-I becomes undetectable. When conjugation is blocked (*atg-3*), LGG-1-I is accumulated and the lipidated form is not present. More generally, an increased level of lipidated LGG-1-II reflects an increase in the number of autophagic structures, which can result from both an increase of the autophagic flux or a blockage of autophagosomal maturation. The usage of lysosomal inhibitors can help distinguish between those cases [52]. An alternative approach consists of using GFP::LGG-1-expressing strains. Within these constructs, WB using an anti-GFP allows the identification of the lipidated and non-lipidated forms, and an additional lower band that corresponds to the cleaved GFP (Figure 1J). GFP::LGG-2 can also be used for monitoring autophagy through WB, although it has been less extensively characterized and used. When autophagy is functional, a major band for GFP::LGG-2 is observed along with two minor bands, one slightly higher and one slightly lower, which could correspond to post-translational modifications. The lipidated form has not been strictly identified yet with the non-lipidated form GFP::LGG-2(G130A); only the major band is observed [23]. Similarly to LGG-1, a cleaved GFP appears upon formation of the autolysosome, and is very convenient for measuring the autophagic flux. For example, in a context where the formation of the autolysosome is impaired, the amount of the cleaved GFP is decreased, while GFP::LGG-1-I and GFP::LGG-1-II forms are increased.

If WB has been predominantly performed on LGG-1 and LGG-2, the quantification of known cargoes and receptors of autophagy also has been punctually used. In particular, the amount of the aggrephagy cargo PGL-3 and the receptor SQST-1 have been used as indicators of autophagy activity by WB [7,53].

Western blotting approaches are useful to evaluate and quantify autophagy in different contexts. They are also relatively easy to perform, but some limitations should be mentioned. Since LGG-1 expression highly depends on the developmental stage, the population of worms or embryos needs to be synchronized, and the material must be in sufficient quantity. Additionally, the sensitivity of this technique is limited, and subtle changes of autophagic flux state might be not observed. In particular, a modification of the autophagic flux limited to a specific tissue could potentially be very difficult to detect with this technique. 

#### 2.2.2. Monitoring Autophagy Genes Expression by RT-qPCR

While the post-translational modification of several autophagy proteins is very important for autophagy, analyzing the transcriptional level could be a good indication of an autophagy induction/repression. Measuring the transcription level of autophagy genes by RT-qPCR has been an efficient way to detect the induction of autophagy. In the context of starvation or the inhibition of the LET-363/TOR signaling pathway, this technique revealed an increase of mRNA levels of several autophagy genes (*lgg-1*, *atg-18*…). This approach allowed the identification of new actors involved in the regulation of autophagy gene expression in *C. elegans*, and particularly the transcription factor HLH-30, ortholog of mammalian TFEB [54]. In a similar way, in the long-lived insulin receptor *daf-2* mutants, mRNA levels of some autophagy genes are increased, which supports the link between autophagic activity and lifespan expansion [55]. For this technique, the normalization of gene expression to multiple control genes is mandatory (for example *pmp-3*, *cdc-42*) [56,57]. RT-qPCR is very sensitive, and can be performed on many genes in the same experiment even with small samples, so it represents an interesting complementary tool.

### 2.3. Modifying Autophagy

In order to study the links between autophagy and cellular or developmental processes, it is essential to analyze the effects of a modification in the autophagic flux. Through using either genetic approaches or drug treatments, *C. elegans* allows the study of the consequences of an induction or a blockage of autophagy throughout a whole organism.

#### 2.3.1. Genetic Approaches

Yoshinori Ohsumi identified *Atg* genes by genetic screens in *Saccharomyces cerevisiae*, for which he was awarded the 2016 Nobel Prize in Physiology or Medicine. The large majority of those genes have a single ortholog in *C. elegans*, and are involved in autophagy process (Table 1). For example, one of the first autophagy genes characterized in *C. elegans* is *bec-1* [31]. The usage of RNAi to deplete BEC-1 causes defects in dauer formation and extension of the lifespan [31]. Thanks to the international *C. elegans* Gene Knockout Consortium and the Japanese National BioResource Project, numerous knocked-out mutants in autophagy genes are available. Noticeably, additional autophagy genes have been discovered by screening in *C. elegans*. Zhang et al. performed a non-lethal genetic screen to identify mutants for deficiency in the degradation of autophagy aggregates during embryonic development [6,9]. The newly identified genes involved in this autophagy process have been named *epg* (ectopic PGL granules). The *epg* genes are either distantly related to yeast *ATG* genes or have no yeast counterparts [12,14,15]. Numerous new tools such as mutants and fluorescent reporters have been generated by these studies. More recent RNAi screenings allowed the identification of signaling pathways involved in the control of autophagy. The results of the different screenings and the numerous genetic tools available have made *C. elegans* a powerful model to study the genetics behind autophagy process and its modulation. Indeed, the mutants of diverse autophagy genes involved in different steps of autophagy process, such as impairing or decreasing autophagy, are widely used. Additionally, mutants in the regulation of autophagy (*hlh-30*, *TOR*) and adaptors (*sepa*, *sqst-1*) also have been described [9,12,56,58]. The depletion of some autophagy proteins, such as BEC-1 and LGG-1, can be linked to sterility or lethality occurring during development, which can complicate their study at later stages. Nevertheless, RNAi in *C. elegans* can be performed by feeding and has been successfully used to provoke depletions in larva and adults. In addition, RNAi depletion can be performed in a tissue-specific manner, thanks to the development of a genetic tool [59]. Since *C. elegans* presents highly specialized tissues, tissue-specific autophagy genetic tools can be interesting for studying the involvement of autophagy in very specific processes. While tissue-specific knockout are not possible in classical mutants, a tissue-specific RNAi approach has been developed in *C. elegans*. It consists of using a strain impaired for RNAi processing machinery, then transfected with construct with a tissue-specific promoter, which restores the RNAi process. Intestinal-specific depletion of BEC-1 revealed that autophagy activity in the intestine is essential against *S. typhimurium* infection [33]. The main limit of the RNAi approach could be its efficiency, with some RNAi failing to reliably deplete the target protein. So, it is essential to check whether a specific RNAi has an effect on the amount of protein or mRNA. Temperature-sensitive mutants are generally a good tool to overcome lethality in *C. elegans,* but have not been yet reported for *atg* genes. Particular genetic tricks can be exploited for resolving lethality issues. For example, the *lgg-1(tm3489 maternal)* strain was obtained by the transgenesis of *Plgg-1::gfp::lgg-1*, which is expressed in somatic cells but not in germline cells. Indeed, the expression of GFP::LGG-1 starts when embryos reach 20-cell stage, rescuing the adult sterility and allowing the characterization of the phenotype of early embryos depleted for LGG-1 [23,25]. Altogether, the numerous autophagy-related genetic tools available in *C. elegans* allow an in-depth genetic analysis of pathways and epistasis.

#### 2.3.2. Pharmacological Treatments

In mammalian cells, drugs are commonly used for modifying and also analyzing the autophagic flux. In regards to the links between pathologies (neurodegenerative diseases, cancer) and autophagy, drugs that modulate autophagy activity have a strong potential. Those molecules have various effects, and can be sorted into two main categories: autophagy activators and autophagy inhibitors. Drugs causing starvation or ER stress also increase the induction steps of autophagy, whereas some activators have an effect on later steps of the autophagic process, during the autophagosome maturation. Several inhibitors are effective for the blockage of the induction steps (class III PI3P inhibitors), while others impair the autophagosome’s degradation.

In *C. elegans*, several constraints made the pharmacological approaches less preponderant for modulating autophagy. Indeed, worms present a protective cuticle that reduces the penetration and diffusion of drugs. Additionally, it has been suggested that *C. elegans* metabolism reduces the efficiency of drugs, leading to the usage of higher concentrations of drugs in this model [60]. Lastly, numerous studies on autophagy have been performed on the embryos, whose eggshell is impermeable to most molecules, which complicates pharmacological approaches. Nevertheless, several drug screenings and pharmacological approaches have been validated in *C. elegans* for both inducing and inhibiting autophagy. A drug screen on *C. elegans* showed that fluphenazine, a potent autophagy enhancer, was efficient at reducing the proteotoxicity of ATZ (alpha-1-antitrypsin Z) in both *C. elegans* and a mouse model. In mammals, the accumulation of ATZ is linked to liver disease, as well as associated with hepatic fibrosis and hepatocellular carcinoma [48]. In regard to longevity, both resveratrol and spermidine increase the lifespan of the nematode in an autophagy-dependent way [61,62]. On the other hand, Bafilomycin A1 can be used to block the late steps of autophagy and avoid autophagosome maturation, thus delaying the degradation of autophagy substrates, and simplifying their study. It can be administered by feeding or injection; both approaches showed an effect on autophagy [63,64]. The inactivation of autophagy using inhibitors of type III PI3K, Wortmannin, and 3-methyladenin, causes an hypoxic lethality in the worms [21].

Alternatively, exposure to stress such as starvation or heat-stress are also an option that can be exploited to induce autophagy. Starvation strongly increases the volume of autophagosomes [34], and thermotolerance affects the transcription of autophagic genes, autophagosome numbers, and mitochondrial degradation [20,35,65,66]. The main limitation of using either pharmacological or physical treatments is the potential secondary effects on various other cellular processes. Additionally, autophagy induction or inhibition caused by treatments could be a secondary effect of the potential disruption of an unrelated process. In summary, this approach is powerful for performing mechanistic analyses, but should be used cautiously for interpreting physiological processes.

## 3. Tools to Monitor Selective Autophagy

Depending on the cargoes that are sequestered and degraded, autophagy has been qualified as bulk or selective. Selective autophagy specifically recognizes and engulfs proteins or organelles through autophagy receptors that mediate the interaction with ATG8/LC3 proteins. Autophagy adaptors could have a function during selective autophagy, but have no roles on cargo recognition, and are not degraded during the process [67]. It can be sometimes difficult to distinguish between bulk and selective autophagy because they mainly share the same autophagy machinery and can contain similar organelles, such as mitochondria or ER. This part concentrates on two selective processes: the aggrephagy and the mitophagy, for which *C. elegans* has been efficiently used for in vivo analyses.

### 3.1. Aggrephagy

Autophagy is an efficient mechanism for the degradation of proteins, and in particular of aggregates, through a selective process called aggrephagy. During *C. elegans* embryogenesis, several aggregate-forming proteins are selectively removed by aggrephagy [53].

#### 3.1.1. P Granules Degradation through Aggrephagy

P granules are ribonucleoprotein aggregates synthesised in germline and transferred to offspring through oocyte. P granules were proved to have a function in germ cell determination during embryogenesis, and are restricted to germline precursor cells, while the P granules components in somatic cells are quickly degraded [7,68]. Two types of P granules components, the proteins PGL-1 and PGL-3, are selectively degraded through aggrephagy, and in autophagy mutants, P granules-like (PGL) structures accumulate until the larva stage. By a genetic screening, the SEPA-1 protein was identified as an important factor for P granules degradation. SEPA-1 binds with both PGL-3 and LGG-1 for cargo recognition in this process, and SEPA-1 itself is also degraded by autophagy [7]. Western blot analysis showed that the *sepa-1* mutant caused the accumulation of PGL granules. Immunostaining assay revealed the co-localization of SEPA-1 with both PGL granules and LGG-1 dots, which indicated that the SEPA-1 protein may be a bridge molecule in mediating P granule selective autophagy. Then, the co-immunoprecipitation confirmed the interaction of SEPA-1 with PGL granules and LGG-1. These data demonstrated that SEPA-1 is a receptor for selective autophagy in P granules degradation.

Several tools have been generated to allow the analysis of aggrephagy in the embryo. P granules can be analyzed by EM, immunofluorescence or Western blot on embryo extract, and transgenic worms expressing GFP::PGL-3 or GFP::PGL-1 in germline allow in vivo observation. In wild-type embryos, GFP::PGL-1 granules are detected only in germ cell precursors, while in autophagy mutant embryos (*lgg-1*, *atg-3*, *atg-4.1*, *atg-7*, *unc-51*), GFP::PGL-1 granules accumulate in the whole embryo. Using this selective aggrephagy process, Zhang et al. performed very powerful genetic screens that permitted the identification of the EPG proteins [6,9] (see also Section 2.3.1).

#### 3.1.2. Poly-Q Aggregates

Due to overexpression or misfolding, some proteins form aggregates within the cell that must be then degraded. In mammalian cells, proteins containing polyglutamine (polyQ) are prone to form aggregates when the number of glutamine residues is above the normal length, which leads to a cellular toxicity. It has been reported that the formation of some polyQ aggregates can be responsible for neurodegenerative diseases [69], and that autophagy is a major pathway to degrade polyQ aggregates within the cell [70,71]. Thus, polyQ reporter proteins have been used for the study of autophagy in *C. elegans* as a substrate to reflect the autophagy activity. A series of transgenic strains have been generated that express a reporter GFP protein fused to different length polyQ tracts in various tissues [72,73,74,75]. Diffuse GFP fluorescence indicates that the polyQ constructs are not aggregated, while GFP clusters correspond to polyQ aggregates, allowing the tracing of polyQ aggregates in vivo by time-lapse microscopy. Interestingly, it has been shown that huntingtin-like polyQ aggregates can be extruded out of neuron cells, and that the extrusion is increased when autophagy is blocked [76]. Moreover, polyQ aggregates remain insoluble after worm lysis, and can be detected by WB [72]. The capacity of polyQ to form aggregates is correlated with the number of glutamines, although it is also affected by aging or stress. The inactivation of autophagy genes affects the amount of polyQ aggregates and increases their toxicity [77,78]. The polyQ aggregates can also be directly detected and quantified by electron microscopy. In *C. elegans*, it has been shown that polyQ aggregates formed during aging were associated with ubiquitination. In mammalian cells, p62 functions as an autophagy receptor for recognizing ubiquitinated protein aggregates, and SQST-1, the homologue of p62 in *C. elegans*, mediates autophagy during embryogenesis [9]. Similarly to p62 in mammalian cells, SQST-1 aggregates also accumulate in autophagy deficiency contexts in both embryo and larva stages. However, whether SQST-1 is involved in the degradation of polyQ aggregates has not been studied in *C. elegans*.

### 3.2. Selective Degradation of Mitochondria by Autophagy

Mitophagy is one of the most studied of the selective autophagy processes. Studies in yeast and mammalian cells have revealed that mitophagy is an important mechanism for mitochondrial quality control, and its impairment has been linked with pathologies such as cancers as well as neurodegenerative and mitochondrial diseases. In *C. elegans*, the study of mitophagy was mainly focused on its role in eliminating paternal mitochondria [19,24]. More recently, some studies have started to exploit the advantages of *C. elegans* to explore the roles of mitophagy in the context of aging or other stress conditions [65,66]. Since mitophagy and bulk autophagy share a common machinery, the tools described previously (see Section 2) are useful to study mitophagy in *C. elegans*. However, it is becoming crucial to develop a series of methods that are able to specifically discriminate mitophagy from bulk autophagy. We briefly describe here the recent tools that allow the analysis of the mitophagy flux.

#### 3.2.1. Mitophagy of Paternal Mitochondria

In most metazoans, mitochondrial DNA (mtDNA) is maternally inherited [79], and paternal mitochondria are eliminated by diverse mechanisms that have been studied in several model animals. These degradative mechanisms can occur before or after fertilization, and result in the clearance of the whole paternal mitochondria, including its mtDNA. The presence of autophagy markers around sperm material in the fertilized oocyte suggested that autophagy may be involved in this process. Indeed, studies in *C. elegans* have shown that upon fertilization, the paternal mitochondria and other sperm specific membranous organelles (MOs) are selectively removed by autophagy during a process called allophagy [19,24]. In vivo imaging and immunofluorescence are the most widely used approaches to analyze allophagy. The main substrates of allophagy are paternal mitochondria and MOs, which can be labelled by the antibodies 1CB and SP56 [80,81]. In the absence of a specific antibody for paternal mitochondria, sperm mitochondria are labelled with a mitochondrial-targeted GFP or through using a dye. For instance, applying mitoTracker or tetramethylrhodamine, ethyl ester (TMRE) to males before mating allows researchers to stain and trace paternal mitochondria in the embryo [19,24]. Tracing mtDNA has been also used for monitoring paternal mitophagy through the labelling of males with the nucleic acid dye SYTO11 [82]. Alternatively, a specific genetic strain can be used to detect paternal mtDNA by PCR. In this particular heteroplasmic strain, a part of mtDNA harbors the uaDf5 deletion that is easily discriminated from wild-type mtDNA [83]. Sperm mitochondria carrying the uaDf5 mtDNA are normally quickly degraded upon fertilization, but persist in the progeny when autophagy is blocked in the oocyte [19,24]. Autophagosomes are analyzed with tools described above (see Section 2.1) using LGG-1 and LGG-2 antibodies or fluorescent reporters as well as EM. In early embryos, the presence of double-membrane vesicles engulfing paternal mitochondria was revealed by EM analysis, further demonstrating mitophagy [24]. Immunostaining analyses demonstrated the co-localization of paternal mitochondria and MOs with LGG-1 and LGG-2 soon after fertilization until 8-cell stage. In *unc-51*, *atg-5*, *atg-7,* and *atg-18* autophagy mutants, the number of LGG-1/2 dots strongly decreased and paternal mitochondria persisted. A recent study has identified the prohibitin PHB-2 as a novel mitophagy receptor involved in the degradation of paternal mitochondria in *C. elegans* [84]. Interestingly, analysis of allophagy revealed that despite the formation of LGG-1 and LGG-2 double-positive autophagosomes, LGG-1 and LGG-2 participate differently in the process. Time-lapse assay with a tandem fusion protein GFP::mCherry::LGG-1, EM, and Western blot experiments were used to characterize the autophagic flux. In the *lgg-1* null mutant, the formation of autophagosomes is blocked, whereas the *lgg-2* null mutant only delays the formation of autolysosomes during allophagy. Further studies using a yeast two-hybrid screen and co-localization in vivo revealed that LGG-2 interacts with the HOPS protein VPS-39 to facilitate the fusion between autophagosomes and lysosomes [25].

Allophagy is essential for the elimination of sperm materials, but it is probable that other degradative mechanisms involving proteasome or other proteins could also be involved in this process [85]. A recent study reported that the mitochondrial endonuclease G, encoded by the gene *cps-6*, is involved in the breakdown and aggregation of sperm mtDNA within the mitochondrial matrix after fertilization, but before autophagosome engulfment. The mutant of paternal *cps-6* slows down the internal breakdown of paternal mitochondria, and in turns leads to a delay of paternal mitochondria degradation [82].

#### 3.2.2. Mitophagy in Stress Conditions

In normal conditions, basal mitophagy in *C. elegans* is kept at a low level. Many stresses can cause mitochondrial damages, and the dysfunction of mitochondria could result in cell toxicity linked to the increase of ROS level or the triggering of the apoptotic cascade. However, if a severe mitochondrial stress leads to mitochondrial dysfunctions and cellular toxicity, a mild mitochondrial stress could activate a beneficial adaptive response and extend lifespan in *C. elegans* [35,86]. The induction of mitophagy is essential to remove dysfunctional mitochondria, maintain cellular homeostasis, and play a protective role in stress conditions. As a model animal, *C. elegans* has been used to analyze the effect of various mitochondrial stresses, paving the way to study the regulation and the roles of mitophagy on stress tolerance. This part briefly presents the tools that are used to monitor and trigger mitophagy.

**Figure 2:**
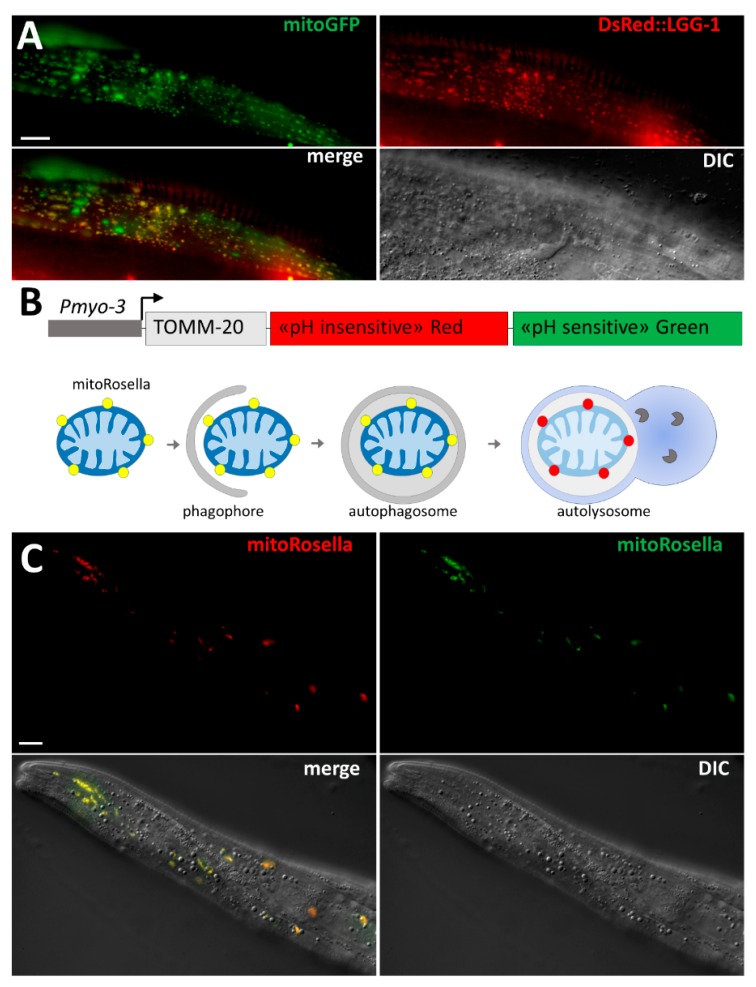
Tools for the study of mitophagy in *C. elegans*. (**A**) In vivo confocal images of mitophagy in the body wall muscle cells of an adult worm. After heat stress (37 °C for 2 h), mitophagy is visualized by co-localization between fragmented mitochondria (mitoGFP in green) and autophagic structures (DsRed::LGG-1); (**B**) Schematic diagram of mitoRosella fluorescent protein biosensor under the control of the muscle-specific promoter *myo-3*. MitoRosella is a chimeric protein-containing fragment of TOMM20, which leads to mitochondrial localization, the pH-stable DsRed fluorescent protein, and the pH-sensitive GFP. During mitophagic flux, mitochondria turn from yellow (DsRed and GFP) to red in autolysosome, due to the quenching of the GFP signal; (**C**) Confocal images of mitoRosella in body wall muscle cells of an adult worm in standard condition (20 °C). All mitochondria are fluorescent for GFP and DsRed, indicating that the basal level of mitophagy in muscle is very low. Scale bar = 10 μm.

The original method used to identify mitophagy was EM, which has been widely used in yeast, mammalian cells, and *C. elegans*. The anatomy of mitochondria is very characteristic, and EM analysis is a powerful approach for the identification of phagophores and autophagosomes that are sequestering fragmented mitochondria [87]. However, EM has not been extensively used in *C. elegans* for characterizing mitophagy in stress conditions, probably because it is time-consuming and costly. As a result, in vivo approaches that are easy to carry out in *C. elegans* and enable the analysis of numerous samples have been preferred, such as monitoring the co-localization of fragmented mitochondria with autophagosomes marked by fluorescence (Figure 2). Thanks to the genetic tools developed in *C. elegans*, researchers have generated transgenic worms expressing fluorescence proteins for observation in living animals. For instance, transgenic worms expressing mitochondria-targeted GFP and DsRed::LGG-1 have been used to detect the co-localization of mitochondria and autophagosomes indicating mitophagy events (Figure 2A) [66]. However, the overexpression of DsRed::LGG-1 may form aggregates, and this transgenic strain should be used carefully and with rigorous controls. Alternatively, the tandem fluorescence protein mitoRosella has been developed to monitor in vivo the mitophagic flux (Figure 2B). During the last step of autophagy, the autophagosomes fuse with lysosomes, which allows for the formation of an acidic compartment, the autolysosome, favorable for the degradation of cargoes. MitoRosella is a biosensor containing a fast maturing pH-insensitive DsRed and a pH-sensitive GFP addressed to the mitochondria. Thus, measuring the ratio between GFP and DsRed fluorescences allows researchers to monitor the fusion of mitochondria-containing autophagosomes with lysosomes [66]. Although mitoRosella has been efficiently developed for monitoring the mitophagic flux in the body wall muscle cells (Figure 2C), its heterogeneous level of expression between individual muscle cells may affect the morphology and the homeostasis of the mitochondrial network.

Upon stress conditions, mitophagy is generally considered to be a degradative process that enables researchers to recognize and selectively remove fragmented and dysfunctional mitochondria. When mitophagy is deficient, damaged mitochondria accumulate in the cells, which results in major consequences on the homeostasis of the cell. So, it can be useful to measure the reactive oxygen species (ROS), ATP, and cytoplasmic Ca^2+^ levels, as well as the oxygen consumption, which are all indicators of the mitochondrial status and could reveal an induction or a defect of mitophagy. Similarly, the analysis of the mitochondrial morphology is informative because mitophagy is often accompanied by mitochondrial fission [88,89]. Transgenic worms expressing mitoGFP or GFP fused with mitochondrial protein such as DCT-1 or a fragment of the yeast TOM70 have been efficiently used to detect mitochondrial morphology in specific tissues [66,90]. During mitophagy in *C. elegans*, the fragmentation of mitochondria can be visualized using P_myo-3_::mtGFP worms in body wall muscle cells, or MitoTracker in numerous tissues. The quantification of mitochondrial fluorescence is also informative, because a reduced amount of mitochondria is one of the features of active mitophagy [66,91].

Since the basal level of mitophagy is very low in *C. elegans*, the use of mitochondrial stress often has been a necessity to better understand the mechanism and physiological function of mitophagy. Drugs that cause the loss of mitochondrial potential (CCCP) or produce oxidative stress in mitochondria (paraquat) are also powerful mitophagy inducers in *C. elegans* [66]. Recent studies revealed that urolithin A, a natural compound belonging to ellagitannins, could induce mitophagy and autophagy in both mammalian cells and *C. elegans*, and prolong the nematode lifespan [91]. Heat stress has been efficiently used to induce mitophagy in *C. elegans*, but other types of autophagy have not been excluded. Finally, the facility to carry out genetic approaches in *C. elegans* could be a powerful means of inducing mitophagy. For example, in *C. elegans*, a partial depletion of frataxin, a main protein of the Fe-S-cluster-containing complex, causes an iron-depletion stress in mitochondria that induces mitophagy [65]. Alternatively, the blockage of mitophagy can be achieved by knocking down either the autophagy general machinery or mitophagy-specific genes. For instance, the mutated form of BEC-1, which belongs to nucleation PI3K complex, blocked mitophagy in *C. elegans* [66], while other studies in Hela cells indicated that BECN1 is dispensable for CCCP-induced mitophagy [92]. Recent studies confirmed that the homologs of Parkin and PINK1 (PDR-1/PINK-1), two well-studied proteins critical and specific for mitophagy in mammalian cells, are also involved in mitophagy in *C. elegans* [66,93]. The deficiency of *pdr-1*/*pink-1* specifically blocked CCCP, paraquat, or heat stress-induced mitophagy in *C. elegans,* and also reduced the lifespan of long-lived animals. Recently, DCT-1 protein, which contains a classic LC3-interacting region (LIR) motif and can interact with LGG-1, has been identified as a receptor for mitophagy in *C. elegans* [66].

## 4. Conclusions

Although this review mainly focuses on macroautophagy, other autophagy pathways, such as chaperone-mediated autophagy and microautophagy, should not been ignored. However, their implications in the physiology of *C. elegans*, has not been yet analyzed. Since macroautophagy is a complex and dynamic process, each method for studying autophagy presents some limitations. In fact, the combination of different approaches is essential when analyzing autophagy to minimize the drawbacks. In regards to recent publications, it is clear that new methods for monitoring autophagy will be rapidly developed to improve and optimize the currently available assays.

If autophagy is a key mediator for cell metabolism, it is now clear that it is acting together with the other degradative mechanisms, and particularly the ubiquitin-proteasome system. As mentioned previously, proteasome may contribute to paternal organelles degradation together with allophagy. Moreover, a recent study identified a complimentary way to analyze autophagy for the elimination of protein aggregates and organelles [67]. Under stress conditions, adult *C. elegans* neurons could extrude vesicles, called exophers, which contain protein aggregates and mitochondria [76]. Both mitophagy and exopher-genesis are involved in proteostasis and mitochondria quality control, and may contribute to relieve neurodegeneration.

## Figures and Tables

**Table 1:** Autophagy genes in *C. elegans*, and homologs.

*C. elegans* Genes	Mutant Alleles or RNAi	Mammalian Homologs	Yeast Genes	References
*atg-2*	*bp576*	*ATG2*	*ATG2*	[6]
*atg-3*	*bp412/RNAi*	*ATG3*	*ATG3*	[7]
*atg-4.1*	*bp410*	*ATG4A, ATG4B*	*ATG4*	[2]
*atg-4.2*	*tm3948*	*ATG4C, ATG4D*	*ATG4*	[2]
*atg-5*	*bp546/RNAi*	*ATG5*	*ATG5*	[8]
*atg-7*	*bp422/RNAi*	*ATG7*	*ATG7*	[7]
*atg-9*	*bp564/RNAi*	*ATG9*	*ATG9*	[6]
*atg-10*	*bp421, bp588/RNAi*	*ATG10*	*ATG10*	[7]
*atg-16.1*	*gk668615d*	*ATG16L1*	*ATG16*	[8]
*atg-16.2*	*bp636, ok3224*	*ATG16L2*		[8]
*atg-18*	*gk378/RNAi*	*WIPI1, WIPI2*	*ATG18*	[9,10]
*bec-1*	*ok691, bp613, ok700/RNAi*	*BECN1*	*VPS30/ATG6*	[10,11]
*epg-1*	*bp414*	*KIAA0652*	*ATG13*	[12]
*epg-2*	*bp444/RNAi*	*?*	*VPS34*	[9]
*epg-3*	*bp405/RNAi*	*VPM1*	*VPS34*	[9]
*epg-4*	*bp425/RNAi*	*EI24*	*VPS34*	[9]
*epg-5*	*tm3425/RNAi*	*EPG5*	*VPS34*	[9]
*epg-6*	*bp242*	*WIPI3, WIPI4*		[6]
*epg-7*	*tm2508*	*FIP200*	*VPS34*	[13]
*epg-8*	*bp251*	*ATG14*	*ATG14*	[14]
*epg-9*	*bp320*	*ATG101*	*VPS34*	[15]
*let-363*	*h98/RNAi*	*TOR*		[16,17,18]
*lgg-1*	*tm348/RNAi*	*GABARAP*	*ATG8*	[7,9,18,19,20,21,22,23,24,25]
*lgg-2*	*tm5755/RNAi*	*LC3*	*ATG8*	[22,24,26]
*lgg-3*	*RNAi*	*ATG12*	*ATG12*	[7,22]
*pgl-3*	*bp439*	*?*		[12]
*rab-7*	*ok511/RNAi*	*RAB7*		[19,23,24,25]
*sepa-1*	*bp456*	*?*		[9,12]
*sqst-1*	*ok2892*	*SQSTM1/p62*		[13]
*unc-51*	*e369/RNAi*	*ULK1*	*ATG1*	[18,27,28]
*vps-34*	*h741*	*VPS34*	*VPS34*	[29]
*vps-39*	*tm2253*	*VPS39*		[25]
*vps-41*	*ep402*	*VPS4*		[25]

**Table 2:** List of autophagic reporters and targets of antibodies in *C. elegans*.

*C. elegans* Protein	Tools	Localization Pattern	References
LGG-1	GFP, DsRed, GFP::Cherry, Cherry, mRFP; antibody	Puncta	[9,25,28,31,40,48]
	GFP::LGG-1(G116A)	Diffuse	[25]
LGG-2	GFP; antibody	Puncta	[24]
	GFP::LGG-2(G130A)	Diffuse	[23]
BEC-1	GFP, mRFP	Puncta/Patch	[28,38]
ATG-4.1	GFP	Diffuse	[2]
ATG-9	GFP	Diffuse	[6]
ATG-18	GFP	Puncta	[47]
EPG-1	GFP	Diffuse	[12]
EPG-2	Antibody	Puncta	[9]
EPG-3	GFP	Diffuse	[9]
EPG-4	GFP	Diffuse	[9]
EPG-5	GFP	Diffuse	[9]
EPG-6	GFP	Diffuse	[6]
EPG-7	GFP	Puncta	[13]
EPG-8	GFP	Diffuse	[14]
LMP-1	GFP	Puncta	[11]
SEPA-1	GFP, RFP; antibody	Puncta/Patch	[7,12]
SQST-1	GFP	Puncta/Patch	[9]
PGL-1	GFP; antibody	Puncta/Patch	[7]
PGL-3	Antibody	Puncta/Patch	[7]
